# Spatial disparity and associated factors of diarrhea among under-five children in Rwanda: a multilevel logistic regression analysis

**DOI:** 10.1186/s12887-024-04748-5

**Published:** 2024-04-24

**Authors:** Abiyu Abadi Tareke, Sebwedin Surur Jemal, Getahun Dejene Yemane, Hamdi Fekredin Zakaria, Enyew Woretaw Shiferaw, Anaclet Ngabonzima

**Affiliations:** 1 West Gondar Zonal Health Department, Amref Health Africa, Gondar, Ethiopia; 2 Department of Statistics, College of Natural and Computational Science, Mizan-Tepi University, Tepi, Ethiopia; 3 Department of Epidemiology and Biostatistics, School of Public Health, Haramaya University, Harar, Ethiopia; 4 Department of Maternal and Child Health (MCH), West Gondar Zonal Health Department, Gondar, Ethiopia; 5 John Snow, Inc.(JSI) Research & Training Institute, Inc, Washington, DC USA

**Keywords:** Children under five, Rwanda, Diarrheal diseases, Spatial, Multilevel logistic analysis

## Abstract

**Background:**

Diarrhea, defined as three or more loose stool per day, is a major cause of child mortality. Exploring its spatial distribution, prevalence, and influencing factors is crucial for public health decision and targeted interventions. This study aimed to investigate these aspects using 2019 Rwanda demographic health survey data.

**Method:**

A total 7,978 (weighted) under-five children were included in this study. Spatial clustering (hotspots areas) were mapped using ArcGIS and SaTscan software. A multilevel logistic regression model was fitted to assessed factors associated with diarrhea, reporting significance at *p* < 0.05 and a 95% confidence interval.

**Results:**

diarrheal diseases in Rwanda showed a clustered spatial pattern (Moran’s I = 0.126, *p* = 0.001), with the primary cluster in west and north provinces. Under-five diarrhea prevalence was 14.3% (95% CI: 13.55, 15.08). Factors increasing likelihood included maternal age 15–34 years, child age 7–24 months, while full immunization was protective (aOR = 0.74, 95% CI: 0.56, 0.98).

**Conclusion:**

Spatial clustering of diarrheal diseases is found in west and north provinces of Rwanda. Being born to a young mother, being a child aged 7–24 months, being fully immunized, being born to a low-educated mother and belonging to a community having low level education are factors associated with diarrheal diseases in Rwanda. Developing interventional plans based on identified clusters and approaching children based on their immunization status, maternal education and age could be cost-effective in reducing diarrheal diseases in Rwanda. Location based intervention could allow for the efficient allocation of resources by focusing on areas with higher prevalence and need.

## Introduction

Diarrhea, according to the World Health Organization (WHO) [1], is characterized by the discharge of three or more loose or liquid stools per day, or a frequency of bowel movements that exceeds the individual’s normal pattern [2]. Diarrhea is not attributed to the frequent passing of well-formed stools, and the same holds true for breastfed infants who pass loose or “pasty” stools—it is not indicative of diarrhea [3]. Diarrheal diseases result from the incomplete absorption of water from the contents of the intestinal lumen. A reduction in absorption or an increase in secretion causes an accumulation of excess water in the lumen, leading to watery stools and a decrease in stool consistency [4].

Globally, diarrheal diseases rank as the second leading cause of death in children under the age of five. Each year, diarrhoea kills around 525,000 children under five., constituting 9% of all global deaths [5]. In addition, diarrhea is a major cause of malnutrition, making the person more susceptible to future bouts of diarrhea and to other diseases [2]. From 2001 to 2008, WHO surveillance networks found that rotavirus infection accounted for approximately 40% of hospitalizations due to diarrhea in children under 5 worldwide [6]. Severe dehydration and fluid loss were the primary causes of death in most children with diarrhea. However, other factors, like septic bacterial infections, may contribute to a growing percentage of diarrhea-related fatalities [2]. Unsafe drinking water and inadequate sanitation facilities [7], prevalent in Rwanda [8, 9], pose the highest risk factors for diarrheal diseases.

A pooled analysis of sub-Saharan countries’ demographic health surveys revealed a high prevalence of childhood diarrhea (15.3%) [10]. Despite advancements in reducing under-five mortality, childhood diarrhea remains the primary cause of morbidity and mortality in this vulnerable population [11]. Different studies iwere identified factors that are associated with diarreal diseases among under-five children. These factors include the age of the mother or caregiver [12, 13], child’s age [14, 15], immunization [16, 17], mother’s/caregiver’s age [18–20], and wealth status of family [13, 15].

This study aims to address limitations observed in prior research conducted in Rwanda, where traditional binary logistic regression was employed to identify variables associated with diarrheal illnesses [21–23]. A notable drawback of traditional binary logistic regression is its failure to account for the hierarchical structure of the DHS data, specifically the intra-cluster correlation [24]. This lack of consideration can result in skewed model estimations, leading to either overestimation or underestimation of model parameters. To overcome this issue, the current study employs multivariable multilevel logistic analysis, offering improved statistical power and more accurate model estimates for this hierarchical data structure.

Another limitation addressed by this study involves the absence of consideration for the geographic distribution of diarrheal diseases in children aged 0–59 months in previous research in Rwanda. Past studies typically modeled data at the national level without assessing sub-regional localization of disease clustering. To overcome this limitation, the current study employs spatial modeling (SaTScan) to analyze the 2019 Rwanda Demographic Health Survey (RDHS) data. This approach allows for the identification of localized clusters of diarrheal diseases, providing valuable insights for local decision-makers.

Finally, this research draws upon data from the latest national demographic health survey, providing insights into the most recent performance of Rwanda in reducing diarrheal diseases among children under five. The study’s findings reflect the current image of the country toward the prevention of diarrheal diseases among under-five children. Notably, the data used is nationally representative and boasts a large sample size, contributing to robust statistical estimates. This distinguishes it from earlier studies in Rwanda, enhancing the reliability and relevance of the research findings.Therefore, the study aims to assess how these diseases are geographically distributed, quantify their prevalence among different populations, and identify the various factors contributing to their occurrence of diarrheal diseases among children aged 0–59 months in Rwanda using evidence from the 2019 Rwanda demographic health survey (RDHS) data.

## Methods

### Study area, data source and study period

This study utilized data extracted from the Rwanda Demographic Health Survey (DHS), conducted over the period spanning from November 9, 2019, to July 20, 2020. The DHS is a comprehensive and nationally representative survey that gathers essential information on health, population, and socio-economic indicators.

In the context of Rwanda, the administrative structure of the country is delineated into hierarchical levels [25]. At the foremost administrative level, Rwanda is organized into four provinces: northern, southern, eastern, and western Provinces. Complementing these provinces is the capital city, Kigali, which functions as a distinct administrative unit. This initial division forms the basis for regional governance and planning. Further subdivision occurs within each province, with the establishment of 30 districts across the nation. These districts serve as integral administrative units, fostering localized governance and facilitating the implementation of policies tailored to specific geographic regions. The district level is pivotal in addressing regional variations and ensuring effective governance. Delving deeper into the administrative structure, each district is intricately divided into smaller units known as sectors. This tiny division results in a total of 416 sectors throughout the country. Sectors play a crucial role in grassroots administration, allowing for a more localized and targeted approach to issues related to health, education, and socio-economic development [25].

### Sampling technique and study population

The RDHS employed a well-structured sampling approach, using the Enumeration areas established during the 2012 Population and Housing Census of Rwanda as the foundational sampling frame. This approach ensures the representativeness of the survey findings by leveraging the comprehensive and up-to-date information gathered during the national census.

The sampling process adopted a two-staged procedure to capture a diverse and balanced representation of both urban and rural populations. In the initial stage, a total of 500 enumeration areas were carefully selected, with a deliberate allocation of 112 areas in urban settings and 388 in rural places [26]. Moving to the second stage of the sampling process, a systematic approach was applied to select 26 households from each enumeration area resulting 13,000 households. From those 13,000 households all 7,756 (unweighted) living children under age five were interviewed for the presence of diarrhea in the last two weeks preceding the survey time.

In the context of the study at hand, the resulting sample size comprises 7,978 under-five children. Note that this sample is not a mere enumeration but is weighted, meaning that the data is adjusted to account for the complex survey design and to accurately represent the entire population. This careful consideration of the sampling strategy and the subsequent weighting of the sample enhance the validity and reliability of the findings, providing valuable insights into the health and demographic dynamics of under-five children in Rwanda as of 2019.

### Study variables

#### Dependent variable

The study focused on diarrhea as the dependent variable among children under the age of five. The response variable “diarrhea” underwent recoding: Mothers or caregivers who affirmed “yes” in response to the question, “Did the child experience diarrhea in the last two weeks?” were designated with a label of 1, while those responding with “no” were assigned a label of 0 (used as the reference category).

### Independent variables

Factors considered at the individual level for their association with diarrheal cases in Rwanda included the child’s age, child’s sex, immunization status, twin status, maternal age, maternal educational status, wealth status, perceived distance from health facilities, types of latrine, sources of drinking water, maternal working status, number of under-five children, and media exposure. Additionally, community-level factors such as place of residency, community-level education, and community-level poverty were also taken into account.

### Operational definition

#### Media exposure

Composite variable created by considering the three media sources: reading the newspaper, listening to the radio, and watching television. If a respondent is exposed to at least one of the three media sources, she is categorized as “exposed.” And If a respondent is not exposed to any of the three media sources, she is categorized as “not exposed” and coded as “0.” Combining the three media exposure variables helps overcome the reduction in the sample size that might occur if each media exposure variable were considered separately. For instance, the question about reading a newspaper might only be relevant for educated women, and the question about watching television might only be applicable to women with access to electricity. Combining them allows for a broader inclusion of respondents.

#### Type of latrines

Categories of latrines in this study were classified into two groups. The first group, labeled as “unimproved toilet,” included populations using toilets characterized by flushing to somewhere else, pit latrines without slabs, bucket toilets, hanging toilets, or other types of toilets. The second group, termed as “improved toilet,” encompassed populations using toilets that flushed to a piped sewer system, flushed to a septic tank, flushed to a pit latrine, flushed with an unknown destination, pit latrines with ventilation improvement, pit latrines with slabs, or composting toilets [27].

#### Sources of drinking water

Drinking water sources were categorized based on the type of access in households. Those using piped water inside their dwelling, piped to the yard/plot, from a public tap/standpipe, piped to a neighbor, tube well or borehole, protected well, protected spring, rainwater, tanker truck, cart with a small tank, or bottled water were coded as “improved drinking water.” On the other hand, households relying on unprotected well, unprotected spring, surface water, or other sources were coded as “unimproved drinking water” [27].

#### Perceived distance from health facility

The DHS program assesses caregivers’ or mothers’ perceptions of the distance from a health facility. Specifically, respondents are asked whether they consider the distance to be a “significant problem” or “not a significant problem” when seeking medical advice or treatment for themselves during periods of illness.

#### Child’s immunization status

A child is classified as “fully vaccinated” if they have received one dose of BCG vaccine, three doses each of polio vaccine, pentavalent vaccine (DTP-hepB-Hib), and pneumococcal conjugate vaccine (PCV), two doses of Rotavirus vaccine, and one dose of measles vaccine. Otherwise, if any of these criteria are not met, the child is considered “not fully vaccinated” [28].

#### Community level of poverty

Community poverty level is determined based on the proportion of households assigned to the poorest and poorer wealth index categories. Households falling at the median value and above are classified as having a high poverty level, while those falling below the median value are categorized as having a low poverty level. The median is chosen as the cutoff point due to the skewed distribution of the variables. A similar categorization approach was applied to determine community-level educational status.

#### Community-level of educational status

Educational status at the community level is determined by the proportion of mothers or caregivers with primary education or higher, categorizing as having a “high community level of educational status,” while those community(cluster) without such education are classified as having a “low community level of educational status.”

### Data management and analysis

The key characterstics of the dataset utilized in this study was described using median, table and percent. Data cleaning was conducted using excel. Weighted frequencies are used to account for the unequal representation of different groups in the sample.

### Spatial cluster analysis

Spatial analysis stands as an increasingly significant field within the realm of geographical information systems, showcasing rapid advancements in technologies and diverse applications that play a pivotal role in addressing public health challenges. In the pursuit of comprehending the spatial distribution of diarrhea among children under the age of five, the study employed ArcGIS version 10.8 software, a powerful tool known for its geospatial capabilities.

The methodology involved the utilization of the global spatial autocorrelation model, with the calculation of Moran’s I value. This statistical measure was instrumental in discerning the nature of the spatial pattern of diarrhea prevalence across the study area [29]. Moran’s I value, ranging from − 1 to 1, serves as a key indicator to ascertain whether the occurrences of diarrhea exhibit a dispersed, clustered, or uniformly distributed spatial pattern. A Moran’s I value close to -1 suggests a dispersed spatial pattern, signifying that cases of diarrhea are scattered across the study area. Conversely, a value near 1 indicates a clustered spatial pattern, revealing that instances of diarrhea are concentrated in specific geographic regions. Meanwhile, a Moran’s I value of zero implies a random distribution, suggesting no discernible spatial pattern.

The study employed a purely spatial scan statistic, implemented through SaTScanTM version 9.7, to pinpoint statistically significant hotspot areas related to diarrhea prevalence among the studied population. This specific scan statistic operates on a Bernoulli model, a statistical model tailored for 0/1 event data, where occurrences are represented as binary responses. In the context of this study, the events are classified as either controls (individuals responding “no” to diarrhea) or cases (individuals responding “yes” to diarrhea). The Bernoulli model is particularly suitable for binary data analysis, making it well-suited for scenarios where the outcome of interest, such as the occurrence of diarrhea, is dichotomous. This model allows the identification of clusters or hotspot areas where the occurrence of diarrhea significantly deviates from what would be expected based on random chance.

In this analysis, the purely spatial scan statistic utilized the default setting for maximum-sized clusters, which involved considering 50% of the population at risk. This setting helps in identifying clusters that are geographically compact and have a higher prevalence of diarrhea cases than the surrounding areas. By using this approach, the study aimed to reveal spatial patterns and concentrations of diarrhea cases that may have been otherwise obscured.

By employing such spatial analytical techniques, the study aimed to uncover not only the geographical variations in diarrhea prevalence among children under five but also the potential clustering of cases. This nuanced understanding of spatial patterns is crucial for developing targeted public health interventions, optimizing resource allocation, and tailoring healthcare strategies to specific regions where the burden of diarrheal diseases may be higher. Ultimately, the integration of spatial analysis in public health research enhances our ability to formulate more effective and geographically informed health policies and interventions.

### Mixed model

Multilevel logistic regression analysis was fitted to identify factors associated with diarrhea in Rwanda. Multilevel analysis is useful for nested data like DHS. For instance, there are individual-level characteristics, such as each mother’s education and household wealth status. We expect that the higher the mother’s income and education, the lower the likelihood of diarrhea cases among her children. However, the mother’s individual level characteristics i.e., income and education might be predicted by enumeration area level/community level characteristics like the place of residency, community level media exposure, community level poverty or regions in which they reside.

In DHS data, the scores of individual-level characteristics of children are more likely to be more correlated within clusters or enumeration areas than between other clusters. The similarity of the score of individual characteristics within a cluster violates assumptions of independence for classical regressions. Multilevel analysis can address the lack of independence of the observations while analyzing nested/hierarchical data as RDHS.

Two-level binary logistic regression (i.e., individual and community level) was fitted to identify factors associated with diarrhea. Four models were fitted. Of the four models, the null model (model not including independent variables), also called the random intercept model, was fitted to calculate the extent of cluster variability on diarrhea. Model fit was assessed using different fitness parameters as the Likelihood Ratio test (LLR), deviance, Akaike information criterion (AIC) and deviance information criterion (DIC). The model with the lowest of the four fitness parameters was selected as the best fitted model.

Cluster variability was assessed by using intra-class coefficient (ICC), median odds ratio [30] and Proportional Change in Variance (PCV). ICC measures the percentage variation attributed to the community-level variables, while PCV measures the proportional change in the community-level variance between the null and succeeding models [31]. The Median Odds Ratio (MOR) describes the area-level variance of odds ratio (OR) scale. The MOR is a statistical measure that is determined by calculating the median value of the odds ratio between the area at highest risk and the area at lowest risk. For instance, consider a scenario where the MOR value is 1.2. In this context, if we selected two children having the same personal characteristics one from cluster with high risk of diarrhea and the other one from low risk cluster, relocating the child from the low-risk to high-risk cluster would result in a 1.2-fold increase in the odds of contracting diarrheal diseases. In the absence of any area-level variation, the MOR is equal to 1. The value of ICCs and MORs was estimated from intercept-only models (null model) to examine the presence of clustering and heterogeneity of diarrhea cases between areas. Finally, variables with p-value < 0.2 were considered for multivariate analysis. Adjusted odds ratio with 95% confidence interval (CI) and p-value < 0.05 were used to declare statistical significance.

## Results

### Characteristics of the study population

A majority of the study participants, comprising 6,575 individuals (82.41%), were from rural areas, and a significant portion, specifically 6,543 individuals (82%), were found to be not fully immunized.The child’s median age stood at 29 months, displaying an interquartile range (IQR) spanning from 14 to 44 months. Most children were aged between 25 and 59 months (57.6%), and over half of these children were born to mothers with an educational background at the primary level (Table 1).

**Table 1:** Demographic features of the study participants (*n* = 7,978) in Rwanda for the year 2019

Characteristics	Weighted frequency	Percent
**Age of child in months**		
0–6	923	11.57%
7–12	833	10.45%
13–24	1626	20.39%
25–59	4596	57.6%
**Sex of the child**		
Male	4,022	50.42%
Female	3,956	49.58%
**Child’s immunization status**		
Fully immunized	1,435	17.99%
Not fully immunized	6,543	82.01%
**Maternal age**		
15–24 years	1,261	15.81%
25–34 years	3,918	49.11%
35–49 years	2,799	35.08%
**Maternal educational status**		
None educated	911	11.42%
Primary school	5167	64.76%
Secondary &above	1900	23.82%
**Household’s wealth status**		
Poorest	1,850	23.18%
Poorer	1,537	19.26%
Middle	1,553	19.47%
Richer	1,556	19.5%
Richest	1,482	18.58%
**Distance from health facility**		
Perceived as big problem	1,904	23.86%
Perceived as not big problem	6,074	76.14%
**Types of latrines**		
Unimproved latrine	2,367	29.67%
Improved latrine	5,611	70.33%
**Sources of drinking water**		
Unimproved	1,795	22.5%
Improved	6,183	77.5%
**Being Twin**		
No	7,772	97.41%
Yes	207	2.59%
**Mother’s Current working status**		
No	2,011	25.21%
Yes	5,967	74.79%
**No. of under five children in the households**		
≤ 2 children	7183	90%
> 2children	795	10%
**Media exposure**		
Exposed	6,321	79.23%
Not exposed	1,657	20.77%
**Community level factors**		
**Place of residency**		
Urban	1,403	17.59%
Rural	6,575	82.41%
**Community level educational status**		
Low level	3,924	49.18%
High level	4,055	50.82%
**Community level of poverty**		
Low level	4,232	53.05%
High level	3,746	46.95%

### Prevalence of diarrhea

The prevalence of diarrhea among children under five years in Rwanda was 14.3% (95% CI: 13.55, 15.08). Lower prevalence of the diseases (below the national point prevalence) was noted in the East Province at 11.44% (95% CI: 10.15, 12.87), Kigali at 11.76% (95% CI: 10.00, 13.77), and the Southern Province at 13.24% (95% CI: 11.67, 15.00). In contrast, the North Province had a prevalence of 16.29% (95% CI: 14.31, 18.48), and the West Province had the highest prevalence at 18.55% (95% CI: 16.88, 20.35), surpassing the national point prevalence.Spatial pattern of diarrhea in Rwanda.

**Fig. 1:**
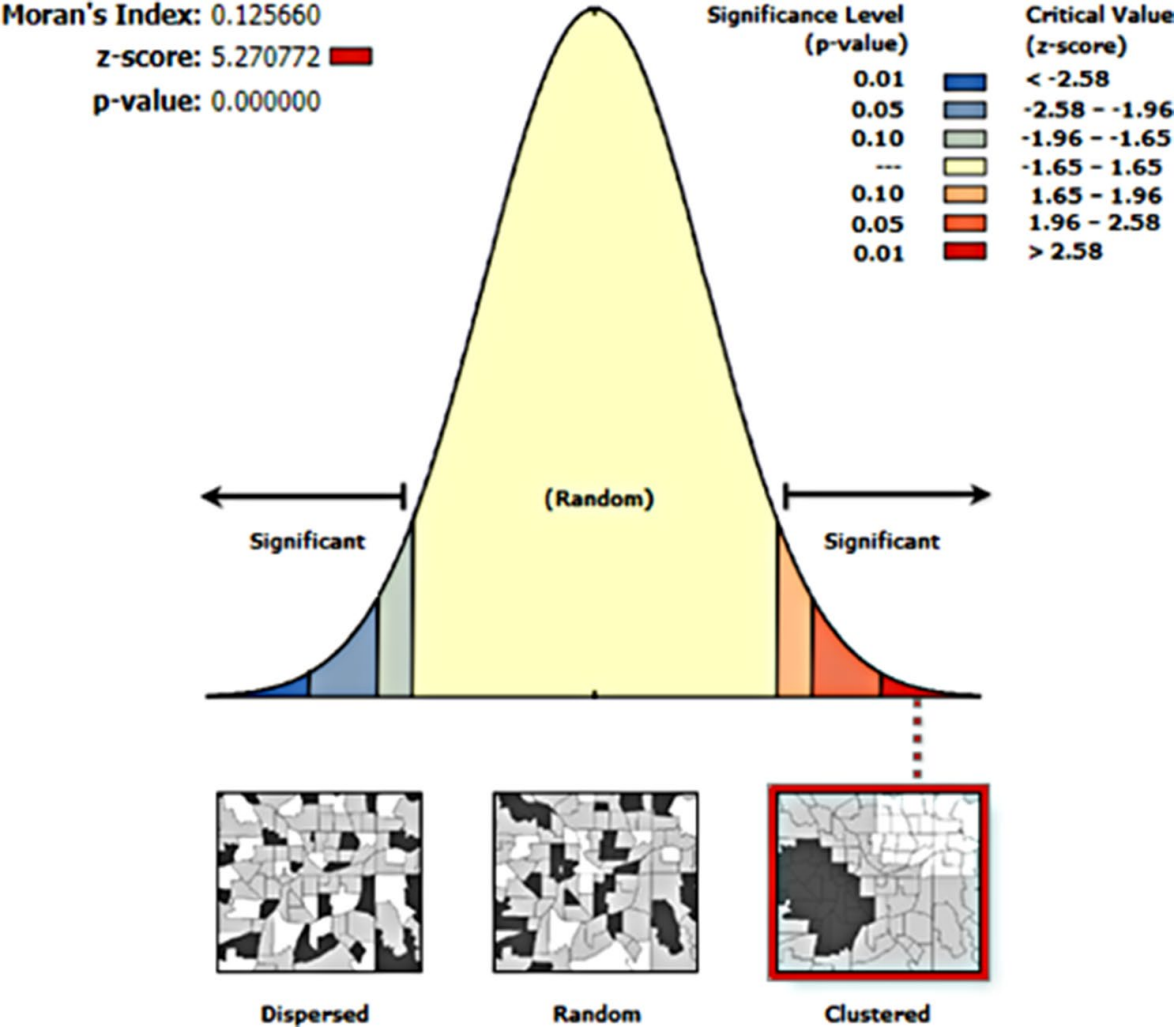
Global Moran’s I analysis to illustrate the spatial pattern of diarrheal diseases among under-five in Rwanda, 2019

The distribution of diarrhea disease among children under the age of five in Rwanda was clustered (global ’Moran’s I = 0.12566, p-value = 0.001) (Fig. 1). Figure 1 presents a map that visually communicates how the occurrences of diarrheal disease among under-five children was distributed across different geographic areas within Rwanda.

### Locations of clustering of diarrheal diseases

**Fig. 2:**
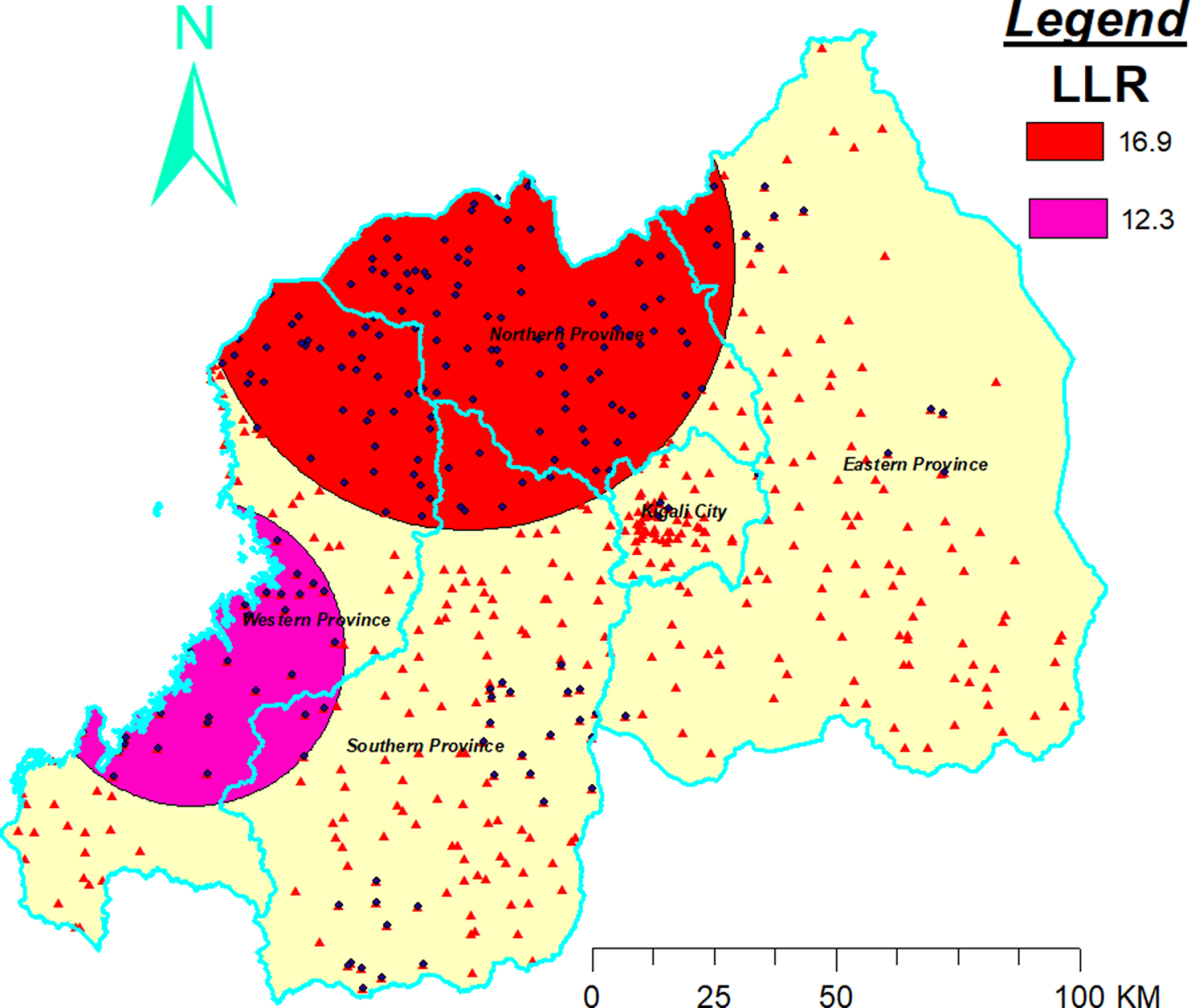
Spatial scan statistics analysis revealing the distribution and hotspots of diarrheal diseases in rwanda for the year 2019

Elevated risk or prevalence of diarrheal diseases among under-five children was observed in north and west provinces of Rwanda (Fig. 2). The primary cluster, the cluster that contains the largest LLR, covers the entire part of the northern province and the north-east part of the western province. The secondary cluster, comprising 216 enumeration areas, is located in the central part of the Western province, centered at (1.475869 S, 29.722419 E), with a radius of 54.72 km.

### Model comparison

Random effects/variance measures included intraclass/cluster correlation (ICC), median odds ratio [30], and proportional change in variance (PCV). The ICC was calculated to ensure intra-cluster variability among study participants. Children from the same cluster are more likely to share common characteristics than children outside the cluster. The result of the intercept-only model (null model) showed the intraclass correlation coefficient was 1.6%. This means that 1.6% of the variation in diarrhea is due to differences between clusters.

The median odds ratio generated from the null model showed the variation of diarrheal disease between clusters. That implies that the cluster in which the child resides also affects the prevalence of diarrheal diseases among under-five children. In this study, the median odds ratio of 1.58 in the null model is interpreted as follows: When pair of children with similar characteristics are randomly selected from different clusters (one from a low prevalence area and the other from a high prevalence area), the individual from the higher-risk cluster is 58% more likely to have diarrheal disease than the individual from the lower-risk cluster. In addition, the proportional change in variance from model III (full model) shows that 4.6% of the likelihood of diarrheal disease among children in Rwanda is due to both individual and community factors (Table 2).

Model three, incorporating both individual and community-level factors, demonstrates the best fit for this data, as evidenced by low DIC and AIC values.

**Table 2:** Comprehensive comparison of models and outputs of fitness parameters for assessing diarrheal diseases in rwanda (2019)

Fitness parameter	Null model	Model I	Model II	Model III
Community level variance	0.2308592	0.2289007	0.1962206	0.2201063
ICC	1.6%	6.6%	5.6%	6.3%
MOR	1.58 [1.44,1.76]	1.57 [1.44,1.78]	1.52 [1.39, 1.71]	1.56 [1.43, 1.76]
PCV [32]	Reference	0.85%	15%	4.6%
**Model fitness parameters**				
Log- likelihood ratio (LLR)	-3153	-2983	-3144	-2979
DIC(-2LLR)	-6306	-5965	-6288	-5958
AIC	6309	5996	6298	5994
BIC	6323	6107	6333	6119

*Note* ICC, intra-cluster correlation; MOR, median odds ratio; DIC, deviance information criterion. The null model is the empty model, the base model without any determinant variable. Model I is adjusted for individual-level factors. Model II is adjusted for community-level factors. Model III is the final model adjusted for both individual and community-level factors

### Factors associated with diarrheal diseases

**Fig. 3:**
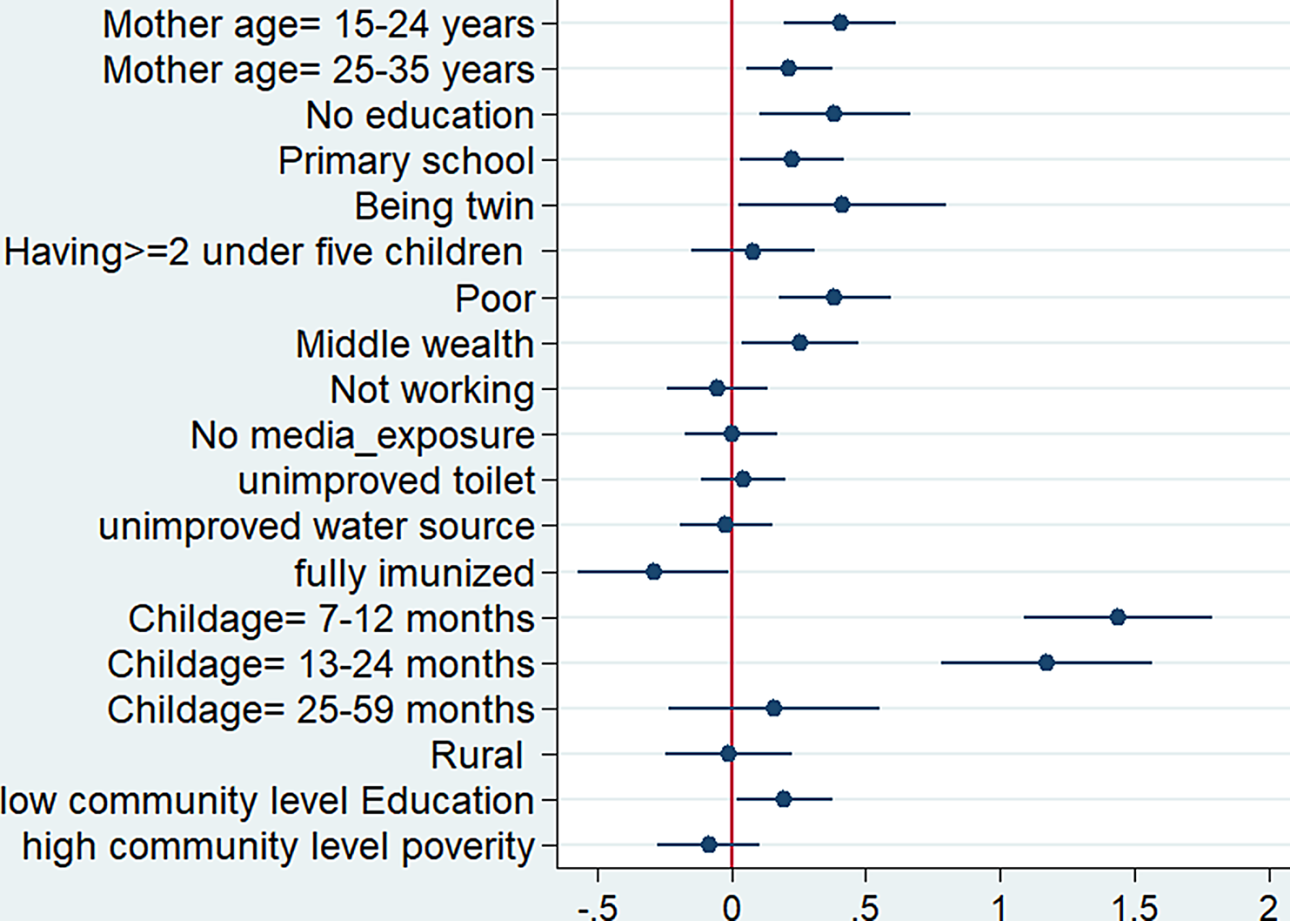
Coefficient plot illustrating the impact of factors of diarrheal diseases among children under-five in rwanda 2019

The coefficient plot in Fig. 3 should be interpreted with attention to the horizontal line, representing the 95% Confidence Interval (CI) of each independent variable’s coefficient. The dot positioned at the center of horizontal line signifies the point estimate of the coefficient. Significance in this context is determined by whether the horizontal line crosses the the vertical line. Variables with a horizontal line that does not cross the vertical line are considered statistically significant. Furthermore, variables to the left of the red vertical line are considered protective, whereas variables to the right of the red vertical line indicate an increased risk of diarrheal diseases.

Therefore, The analysis revealed that several variables were significantly associated with the occurrence of diarrheal diseases among the studied population. These influential factors include maternal age, child’s age, the immunization status of the child, whether the child is a twin, the wealth status of the household, and the educational status at the community level (Fig. 3).

Children whose mothers are in the age groups of 15–24 have a 50% higher risk of experiencing diarrhea compared to those whose mothers are aged between 35 and 49 years. The adjusted odds ratio (aOR) for this association is 1.50, with a 95% confidence interval (CI) ranging from 1.21 to 1.84. Likewise, children born to mothers in the 25–34 age group experience a 24% higher risk of diarrhea (adjusted odds ratio [aOR] = 1.24, 95% CI: 1.06, 1.45) when compared to those with mothers aged 35–49 years.The odds of diarrhea were significantly elevated, showing a 4.21-fold increase (95% CI: 3.00, 5.98), in children aged 7–12 months when compared to the reference group of children aged 0–6 months Furthermore, children aged 13–24 months exhibited a 3.23-fold increase (95% CI: 2.18, 4.79) in the odds of experiencing diarrhea compared to those aged 0–6 months (Table 3).

**Table 3:** Multivariable multilevel logistic analysis of diarrhea diseases among under five years children in Rwanda, 2019

Characteristics	Model I (95%CI AOR)	Model II (95%CI AOR)	Model III (95%CI AOR)
**Maternal age**			
35–49 years	1		1
15–24 years	1.50 [1.22, 1.85]		1.5 [1.21, 1.84] *
25–34 years	1.24 [1.06, 1.45]		1.24 [1.06, 1.45] *
**Child’s age**			
0–6 months	1		1
7–12 months	4.20 [2.96, 5.96]		4.21 [3.00, 5.98] *
13–24 months	3.21 [2.17, 2.75]		3.23 [2.18, 4.79] *
25–59 months	1.16 [0.78, 1.72]		1.17 [0.79, 1.73]
**Immunization status**			
Not Fully vaccinated	1		1
Fully vaccinated	0.74 [0.56, 0.98]		0.74 [0.56, 0.98] *
**Maternal educational status**			
Secondary &above	1		1
None educated	1.55 [1.17, 2.04]		1.46 [1.10, 1.94] *
Primary school	1.25 [1.04, 1.52]		1.25 [1.03, 1.51] *
**Twin**			
No	1		1
Yes	1.49 [1.01, 2.20]		1.51 [1.02, 2.22] *
**No. of under five years children**			
≤ 2 children	1		1
> 2 children	1.08 [0.86, 1.32]		1.08 [0.86, 1.36]
**Wealth index**			
Rich	1		1
Poor	1.46 [1.21, 1.37]		1.46 [1.19, 1.84] *
Middle	1.30 [1.05, 1.60]		1.29 [1.04, 1,60] *
**Mother Working status**			
Working	**1**		1
Not working	0.94 [0.78, 1.39]		0.94 [0.78, 1.14]
**Media exposure**			
Exposed	**1**		1
Not exposed	1.99 [0.84, 1.18]		0.99 [0.84, 1.18]
**Toilet type**			
Improved	**1**		**1**
Not improved	1.04 [0.89, 1.21]		1.04 [0.89, 1.21]
**Source of water**			
Improved	**1**		**1**
Not improved	0.98 [0.83, 1.16]		0.98 [0.82, 1.16]
Community level factors			
**Residency**			
Urban		**1**	1
Rural		1.18 [0.95, 1.46]	0.99 [0.78, 1.73]
**Community level educational status**			
High		1	1
Low		1.23 [0.95, 1.46]	1.21 [1.01, 1.45] *
**Community level poverty**			
Low		1	1
High		1.09 [0.91, 1.29]	0.92 [0.76, 1.11]
Constant	0.045 [0.03, 0.07]	0.11 [0.09, 0.13]	0.45 [0.03, 0.07]

*Note* ®- reference, aOR - adjusted odds ratio, CI- confidence interval

Children born in communities with a low educational status face a 21% higher odds of experiencing diarrhea ([aOR] = 1.21, 95% CI: 1.01, 1.45) compared to their counterparts born in communities with an improved educational status. Children who are fully vaccinated experience a 26% decrease in the odds of diarrhea (aOR = 0.74, 95% CI: 0.56, 0.98) compared to children who are not fully vaccinated.

## Discussion

This study aimed to uncover the spatial distribution and to examine the prevalence of diarrhea among under-five children in Rwanda using data from the most recent demographic health survey of Rwanda. It also examined individual and community-level factors associated with diarrhea diseases among under-five children. The spatial scan statistics identified two clusters (areas having high diarrheal diseases prevalence than expected). The most likely cluster spotted in the north and western provinces of Rwanda (size: 54 km, RR: 1.41 and p-value < 0.0001) might have been due to the high poverty level in those provinces [32]. The access problem to primary health care in western provinces [33] might have caused clustering of diarrheal diseases in tht province. Beyond the poverty and access problems in those areas, other socio-economic, environmental, and healthcare-related variables may contribute to the observed clustering of diarrhea cases. Therefore, better to investigation further to find the cause of clustering in those areas. Provinces engulfed by most likely cluster could be potential outbreak areas for diarrheal diseases. So, public health officials should take all neccesarry measures to halt the high clustering of diarrheal diseases among under-five in those mentioned areas.

The prevalence of diarrheal diseases among under-five children in Rwanda was 14.3% (95% CI: 13.55, 15.08). This is consistent with other studies [12, 34, 35]. However, it is higher than study done in Nigeria (12.7%) [36] and lower than the studies done in Ghana (19.2%) [37], India (25.2%) [38] and Ethiopia (23.1%) [39]. This disparity may be due to differences in sociodemographic characteristics, location, climate, culture, access to water and sanitation, study period and hand-washing culture.

Women in lower age cohorts, compared to the higher age cohorts (35–49 years) and women having twin births, compared to single births, increase the occurrence of diarrhea among under-five children. This result is similar to research conducted in east Africa [12], Uganda [40], and Nepal [41]. Possible explanation is that higher aged mothers have good knowledge and experiences about child health in general and diarrheal disorders in particular [42]. In addition to this higher aged women may have other dauther/son to take care of the their children.

Compared to infants aged 0–6 months, infants aged 7–12 months and 13–24 months have increased odds of acquiring diarrheal diseases. This finding is consistent with other studies [40, 43–46]. The initiation of supplemental feeding after the age of six months may be responsible for the increased prevalence of childhood diarrhea compared to exclusive feeding under six months children. This is might be due to the strong likelihood that children who started supplementary feeding were given contaminated foods, which may have increase the chance of putting diarrhea causing organism to the digestive system. Another reason for the increment of diarrheal diseases after the age of six months might be due to hand to mouth coordinations that increases putting infectious objects to their mouth [47].

Another pertinent finding of this study is the strong correlation between routine immunization and diarrhea occurrence. Being a fully immunized is found protective to diarrhea (aOR: 0.74) compared to a child who was either partially or not vaccinated. This is due to the protective effect of immunization against diarrheal diseases, especially in favor of measles [48] and Rota virus. This study is consistent to a population based cross-sectional study done in urban west Bangladesh provinces [16], besides, in Rwanda, vitamin A, that reduces the prevalence of diarrheal diseases [17, 49] is usually given to under five children, integrated with routine immunisation services.

Moreover, children born to mothers who are not educated or spend fewer years in school have a higher risk of encountering diarrhea than children born to mothers who completed secondary school or more. This finding aligns with a community based crossectional study done using sample size of 13,076 in Nigeria [18]. This correlation might be explained by the sizable positive effect of mothers’ educational attainment in which having higher maternal education is associated with healthy behavior during pregnancy and when raising a child [19]. Besides, the mother’s educational status is a strong predictor of other health measurements like good nutritional status [1, 50, 51] and utilization of healthcare services [20].

Compared to children born to wealthy households, children born to a low-income family increase the likelihood of having diarrhea. Many studies done in Uganda [13], Ghana [15], three east African countries [23] and Rwanda [21]also fortify this evidence. This high odds of diarrheal diseases in economically low householdscan be explained by the fact that economically marginalized families are doubtful to bring their children to health facilities due to concerns of transport fees and the cost of health services. Another possible reason for this finding is that children from poor households are less likely to get a balanced diet and more likely to be malnourished, which precipitates and elongates the duration of diarrhoea [52].

Finally, children born in communities characterized by a low educational status face a 21% higher odds of experiencing diarrhea compared to their counterparts. This finding underscores the influencing role of community-level education in shaping the health outcomes of children. The higher odds of diarrhea in communities with lower educational status suggest a potential link between educational resources, awareness, and health practices. Addressing educational disparities at the community level may prove instrumental in implementing effective public health interventions aimed at reducing the prevalence of diarrhea among under-five children.

### Implications and future research

Our research has implication for Rwanda government minister of health and public health researchers:

The study found a significant clustering of children with diarrheal diseases in west and east provinces of Rwanda. This suggests an opportunity for health authorities and program designers to implement targeted interventions to the west and north provinces, aiming to have rapid reduction of prevalence of diarrheal episodes in the country. This allows for a more efficient allocation of resources to areas with the highest prevalence.

Given the elevated prevalence among under-five children, there is a need for targeted healthcare initiatives in this age group (like health care access, vaccination, educating to their caregivers). The correlation between maternal age and diarrheal diseases in under-five children underlines the importance of maternal health education. Providing information and resources to mothers, particularly those aged 15–24 and 25–34 years, can contribute to better child health outcomes. Additionally, being immunized is found protective to the occurrence of diarrheal diseases. This highlights the importance of comprehensive immunization programs. Strengthening and promoting immunization efforts can be an effective strategy in reducing the burden of diarrheal diseases in the country.

Researcher in the field of public health should consider Conducting longitudinal studies to monitor changes in the spatial distribution of diarrheal diseases over time. Better to conduct investigations that mighly focus on the barriers to healthcare access and utilization, especially in the identified cluster regions. As there might be variation of the relationship between the dependent and independent variables across the study area, we recommend future researchers to conduct local spatial statistical regression like geographically Weighted Regression (GWR). Local spatial regressions are adevantegous in finding factors associated with spatial variation of diarrheal diseases across space and they allows for the examination of spatially varying relationships between variables. This makes it a valuable approach in spatial analysis when relationships are expected to differ across the study area.

### Strengths and limitations of the study

The main strength of this study is that the utilization of substantial nationwide representative data might have increased the statistical power of the study and its generalizability. In addition, in this study the hierarchical nature of the survey was considered by conducting an appropriate statistical multilevel model. This study is based on data collected using crossecrtional method of study, causation cannot be assured. Social desirability bias is inevitable. Another limitation is some study showed that spatial correlation of rate of diseases might occur due to geographic proximity rather than similarities among the diseases [53]. Due to this the spatial correlation of diarrheal diseases rate observed in this study using global moran’s I statistics might have occurred because of spatial proximity rather than similarity of diarreal diseases rate. Hence, we used data from a secondary survey. Other pertinent behavioral and cultural factors are not embodied in this study.

## Conclusion

Spatial clustering of diarrheal diseases was observed in the northwest and Western part of Rwanda. Being born to young mother, being a child aged 7–24 months, being fully immunized, being born to a low-educated mother and belonging to community having low level education were found associated with diarrheal diseases among under-five children in Rwanda. It might also be cost-effective to develop interventional strategies that mainly focus on children aged less than 12 months, those not fully immunized, children from economically marginalized households and children born from young mothers. Developing interventional plans that mainly targeted the identified clusters might help halting diarrheal burden in the country in a fast way.

## Ethical statement

Ethical approval is not required for this study because the authors further analyzed secondary data from the RDHS. These data were granted by registering and stating the aims of the study on the website www.dhsprogram.com. Therefore, we analyzed the anonymized observations, and standard data consent is not required here.

## Data Availability

The datasets and materials are available at www.dhsprogram.com. The minimal data used for this study are available from the corresponding author on reasonable request.

## Abbreviations

AIC: Akaike information criteria
AOR: Adjusted Odds Ratio
BIC: Bayesian information criteria
DIC: Deviance Information Criterion
ICC: Intra-Class Correlation
LLR: Log- Likelihood Ratio
MOR: Median Odds Ratio
PCV: Proportion of Variance Change
CI: confidence interval
AOR: Adjusted odds ratio
EA: Enumeration areas
KM: Kilometer
RDHS: Rwanda Demographic health survey
